# New Zealand Bitter Hops Extract Reduces Hunger During a 24 h Water Only Fast

**DOI:** 10.3390/nu11112754

**Published:** 2019-11-13

**Authors:** Edward Walker, Kim Lo, Sze Tham, Malcolm Pahl, Dominic Lomiwes, Janine Cooney, Mark Wohlers, Pramod Gopal

**Affiliations:** 1 The New Zealand Institute for Plant and Food Research Limited, Auckland 1025, New Zealand; Kim.Lo@plantandfood.co.nz (K.L.); Malcolm.Pahl@plantandfood.co.nz (M.P.); Mark.Wohlers@plantandfood.co.nz (M.W.); 2 The New Zealand Institute for Plant and Food Research Limited, Palmerston North 4442, New Zealand; Kris.Tham@plantandfood.co.nz (S.T.); Dominic.Lomiwes@plantandfood.co.nz (D.L.); Pramod.Gopal@plantandfood.co.nz (P.G.); 3 The New Zealand Institute for Plant and Food Research Limited, Hamilton 3240, New Zealand; janine.cooney@plantandfood.co.nz

**Keywords:** Intermittent fasting, time–restricted eating, bitter taste receptors, appetite, satiety, bitter brake, Amarasate®, water fasting, dietary supplement, calorie restriction

## Abstract

Intermittent fasting improves metabolic and cardiac health. However, increased hunger towards the end of the fasting period may affect compliance and limit its application. Our aim was to determine the effect of anorexigenic agent co-therapy on subjective ratings of appetite during the 16–24 h period of a day-long water-only intermittent fast. Thirty adult men were recruited and required to fast for 24 h from 18:00 h to 18:00 h on the same day of the week for three subsequent weeks. Treatments of either a placebo or one of two doses (high dose; HD: 250 mg or low dose; LD: 100 mg) of a bitter hops-based appetite suppressant (Amarasate®) were given twice per day at 16 and 20 h into the fast. From 18–24 h of the 24 h fast, both the HD and LD treatment groups exhibited a statistically significant (*p* < 0.05) > 10% reduction in hunger. Additionally, the expected lunchtime increase in hunger that was present in the placebo group (12:00 h) was absent in both the HD and LD groups. These data suggest that appetite suppressant co-therapy may be useful in reducing hunger during intermittent fasting, and show that bitter compounds may regulate appetite independently of meal timing.

## 1. Introduction

A healthy diet helps to prevent many chronic diseases prevalent in the Western world, as well as being associated with greater longevity and an improved quality of life [1,2]. Modern excesses in the Western diet have resulted in increased rates of diet-linked disease such as obesity, diabetes, cancer, and cardiovascular disease (CVD) [3,4,5,6], and are associated with unfavorable perceptions of mental state, health, vitality, and physical function [7,8]. To counter these negative health outcomes, dietary interventions are often prescribed as an initial treatment therapy, with many current diet strategies reliant on alterations of macronutrient make up, that typically modify diet composition and impose food choice restriction [9]. While effective if adhered to, more highly restrictive diets are associated with poor compliance that may ultimately result in diet failure [10,11], and suggest the potential for alternative diet strategies. In contrast to many conventionally prescribed diets, intermittent fasting (IF) does not require changes in food choice or diet composition, instead relying on either time-restricted eating [12], recurrent reduction in daily caloric intake [13], or complete water-only fasting days [14], and hence may be an alternative diet option when conventional diets fail. Contemporary research suggests that IF may offer equivalent health benefits compared with classically prescribed diets [15,16,17,18,19,20,21,22,23]. Additionally, IF confers benefits that extend well beyond weight loss. It replicates some health benefits associated with prolonged caloric restriction [16,24], is immunoregulatory [25], effects markers of skin health and dermatology [26,27,28], improves symptoms of arthritis [29], is associated with improved outcomes for cancer patients [30], and enhances glucose regulation post-fast [31]. While diet composition is not modified during fasting, feelings of hunger are increased [32,33], notably during the 16–24 h period of a 24 h water-only fast [34], that may negatively affect dietary restraint and potentially reduce long- and short-term compliance to the diet regime [35,36,37,38]. Appetite suppressant co-therapy may be an avenue to reduce this hunger, increase dietary restraint and hence improve compliance to an IF type diet.

Indeed, traditional medicine records a history of using bitter appetite suppressants to alleviate hunger during times of fasting [39,40], with recent studies showing bitter compounds to be effective anorexigenic agents [41,42]. Hops bitter acid derived compounds reduce body weight, fat mass and improve glucose homeostasis in humans, while also stimulating the release of anorexigenic gastrointestinal (GI) peptide hormones in both human cell lines and in animals [43,44,45]. We hypothesize that consumption of a bitter hops extract will reduce subjective ratings of appetite during a water-only fast day.

In this study, we examined the effect of an extract of New Zealand Hops (Amarasate®) on subjective measures of appetite during the 16–24 h period of a 24 h water-only fast in healthy normal weight men.

## 2. Materials and Methods

### 2.1. Participants

Thirty healthy men (eligibility: age 18–55 year, BMI 20–25 kg/m^2^) were recruited through poster or electronic advertisement in Palmerston North, New Zealand. Potential participants were excluded if they met any of the following criteria: Current participation in a weight loss program or use of weight loss medication, diagnosed diseases of the gastrointestinal (GI) tract, previous GI surgery, diabetes, undergoing food restriction or any condition that may alter body composition within a short period of time, use of nonsteroidal anti-inflammatory drugs, history of ischemic gut, current glucocorticoid use, celiac disease, history of alcohol or drug abuse, recent weight loss/gain (5 kg within the last 6 months), any known medical conditions/medications that may affect the gut or appetite, known allergy, intolerance, or sensitivity to any ingredients in the study product. Participants were also required to be non-smokers, ascertained healthy and able to undertake a 24 h water-only fast by self-report. All subjects provided written informed consent prior to the appetite clinical trial and were allowed to withdraw at any time for any reason. Clinical research was approved by the Southern Human Ethics Trial Board (18/STH/267). All studies were carried out in accordance with the Declaration of Helsinki and the trial was registered at the Australian New Zealand Clinical Trials Registry (ACTRN12619000454178). The trial was conducted at The New Zealand Institute for Plant and Food Research Limited (PFR), Palmerston North, New Zealand, during March and April 2019.

### 2.2. Study Design, Supplements and Protocol

**Figure 1:**
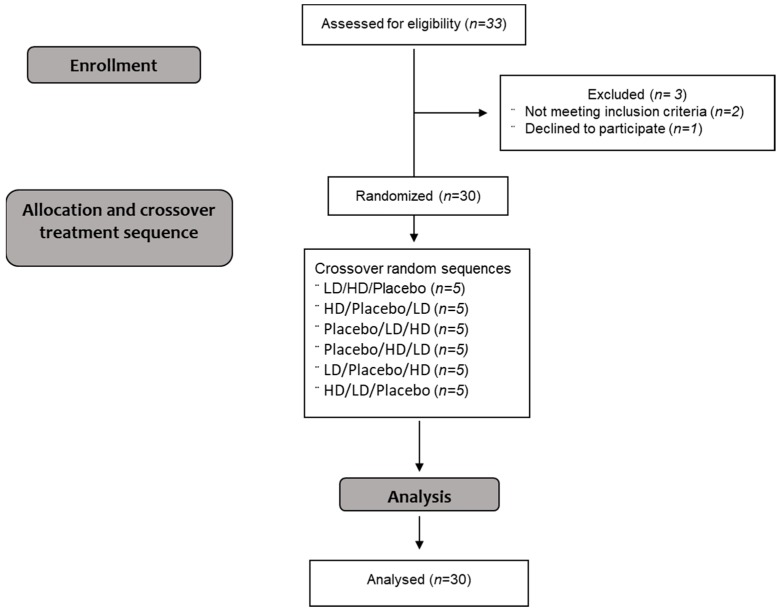
Consolidated Standards of Reporting Trials (CONSORT) flow diagram of the recruitment, enrolment, and random assignment process. HD (high dose—500mg Amarasate^®^), LD (low dose—250 mg Amarasate).

This was a randomized, double-blind cross-over treatment study. It involved two concentrations of the Amarasate hops flower extract suspended in a canola oil excipient and a placebo control, which were protected from gastric acid digestion by encapsulation using a hydroxypropyl methylcellulose (HPMC) size 0 DRCap capsule (DRcaps^TM^, Capsugel, Morristown NJ, USA) and expected to elicit possible anorexigenic effects starting from 30 to 90 min post consumption [46]. The Amarasate hops extract was prepared using a food safe supercritical CO_2_ extraction process using a commercially available New Zealand variety of *Humulus lupulus*. The interventions arms were two formulation matched dosages of the Amarasate extract, high dose (high dose; HD, 500 mg) and low dose (low dose; LD, 200 mg), and a placebo treatment matched to the HD for excipient concentration (Table 1). Treatment capsule weights were 460 mg (HD), 242 mg (LD), and 210 mg (placebo), and were not balanced due to interactions of the hops extract with excipients that affect extract stability and dispersion [47,48]. All treatment groups contained 2 capsules, one given at 16 h and the second given 20 h into the 24 h fast, each containing half the total daily treatment dose. Treatment capsules were produced in a single batch, and compositional stability of treatments was assessed by high performance liquid chromatography (HPLC) analysis of sample capsules set aside immediately pre and post study. Participants were required to attend three study visits on the same day of the week for 3 successive weeks, and were asked to refrain from excessive exercise, activity, and alcohol consumption for 24 h prior to the commencement of the study day. Participants were also asked to keep food diaries for the 72 h prior and 24 h post intermittent fasting days so that adherence to the fasting protocol could be assessed. Randomization was conducted using a 6 × 3 Williams design balanced for order of presentation and carry-over effects. Trial CONSORT flow diagram is shown in Figure 1.

**Figure 2:**
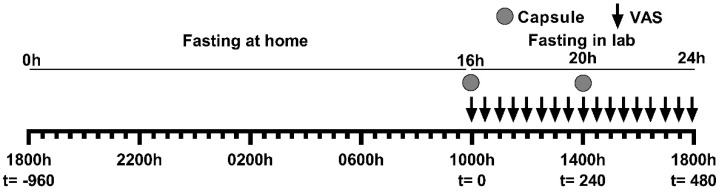
**Study protocol**. Study protocol on days 1–3. At 16 h into the 24 h fast (*t* = 0 min) participants recorded their baseline visual analogue scale (VAS)- based appetite assessments and then consumed a delayed digestion capsule containing either a placebo, a low-dose hops extract or a high-dose Amarasate^®^ hops extract. Every 30 min, VAS-based appetite assessment questionnaires were taken from 16 h until 24 h into the 24 h fast. Then at 20 h into the 24 h fast (*t* = 240 min) a second treatment capsule match to the first was given. • = Capsule given. 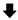 = VAS measurements.

This trial was conducted using a protocol modified from Tinsley et al. 2018 [34] by inclusion of certain methodology described in Thivel et al. 2018 [49], specifically by a setting a prescribed fasting period (18:00 h to 18:00 h), allowing ad libitum eating prior to the commencement of the fast, and allowing water consumption without stipulating restriction during the fast. This study was conducted using paper visual analogue scales (VAS) validated for the subjective assessment of appetite [50,51,52,53,54]. On the evening prior to commencement of the 24 h fast, participants were instructed to eat a typical dinner (i.e., what they would normally eat) until comfortably full before 18:00 h (*t* = −960 min) and then fast overnight with the consumption of water allowed. Participants arrived at the clinical facility at 09:50 h (*t* = −10 min) and were randomly assigned to 1 of 3 treatments after baseline measures were taken. Treatment capsules were given at 10:00 h (*t* = 0 min) and at 14:00 h (*t* = 240 min), presented in an opaque cup and consumed without participants handling the capsules. Appetite measures were taken throughout the day. When capsule administration and VAS assessment occurred at the same time, VAS were conducted immediately before capsule consumption. Water intake was recorded during the 16–24 h fasting period and participants were restricted from drinking immediately prior to and during VAS assessments. Participants were required to stay at the testing facility and remain sedentary until the study day was completed, and no sleeping was allowed (Figure 2).

### 2.3. Appetite Measures

Subjective measures of appetite (i.e., hunger, fullness, satisfaction, thoughts of food (TOF)) and physical comfort (i.e., nausea) were recorded from 16 h to 24 h of the fast, and assessed by participants marking their appropriate subjective feelings on a 100 mm scale. The appetite related VAS questions were as follows: “How hungry do you feel?” (anchored by ‘I am not hungry at all’ and ‘I am as hungry as I have ever been’), “How full do you feel?” (anchored by ‘I am not full at all’ and ‘I am totally full’), “How satisfied do you feel?” (anchored by ‘I am completely empty’ and ‘I cannot eat another bite’), “How much do you think you can eat?” (anchored by ‘nothing at all’ and ‘a large amount’). VAS were measured immediately prior to and then 30, 60, 90, 120, 150, 180, 210, 240, 270, 300, 330, 390, 420, 450, and 480 min after the first treatment capsule. The study was powered to detect a 10 mm (10%) change in VAS relative to the placebo as significantly different, and the primary outcome of this trial was subjective measures of hunger, fullness, and satisfaction.

### 2.4. Characterization of Hops Acid Compounds

Compositional analysis of hops extract and the hops compounds present in the HD and LD treatment capsules was performed using liquid chromatography mass spectrometry (LCMS) with a LTQ linear ion trap mass spectrometer (ThermoFisher, San Jose, CA, USA) coupled to an Ultimate 3000 UHPLC and photodiode array detector (Dionex, Sunnyvale, CA, USA). Chromatographic separation was achieved on a Kinetex 2.6µ EVO C18 100Å (Phenomenex, Torrance, CA, USA), 2.1 × 150 mm ID column maintained at 70 °C. Solvents were (A) water + 0.5% formic acid and (B) acetonitrile + 0.5% formic acid and the flow rate was 800 μL/min. The initial mobile phase, 50% B was ramped linearly to 60% B over 4 min, held for 1 min, then ramped linearly to 75% B over 5 min, then to 100% B over 3 min and held for 1.5 min before resetting to the original conditions. MS data were acquired in the negative mode using a data-dependent LCMS^3^ method. UV-vis detection was by absorbance at 200–600 nm. Sample injection volume was 1 µL.

Extract and treatment capsule samples for LCMS analysis were prepared at a concentration of 1 mg/mL in methanol. An ICE4 hop extract standard (American Society of Brewing Chemists, St. Paul, Minnesota, USA) similarly prepared was used for quantitation of the major hop α- and β- acids.

### 2.5. Statistical Methods

To estimate the required sample size, a power analysis was performed using VAS hunger as the dependent variable. Estimates of variance components were conducted based on data from a previous study examining the effect of a bitter anorexigenic agent on VAS-based hunger assessments [41]. Power estimates showed 30 participants were sufficient to detect a 10% difference in the primary outcome measure of VAS hunger, based on a power level of 0.8 and a dropout rate of 20%. Participant responses collected using VAS were reported as means ± standard error of the means (SEMs). Subjective VAS data were analyzed as a mixed effects model using the Glimmix procedure in SAS 9.4. To take account of the complex cross-over design, panellist and time within panellist were treated as random effects with an auto regressive order 1 (AR1) covariance structure specified to model the residual correlations due to repeated measurements within a session. Visit number, time, treatment, and the treatment by time interaction were included as fixed effects and tested using Type 3 sums of squares with Kenward-Roger degrees of freedom. Residual plots were inspected to confirm that the model assumptions of normality and constant variance were met. Finally, predicted means were produced and pair-wise comparisons amongst treatments within a time point were tested if the treatment by time interaction was statistically significant at the 5% level (*p* < 0.05). As no statistically significant differences were present between any of the treatment groups at baseline time point for any of the appetite measures examined, datasets were then normalized to the baseline reading within a subject and day combination and the full set of analyses repeated. The area under the curve (AUC) for each subject and visit combination was calculated by numerical integration using Simpson’s rule in the sintegral function from the Bolstad2 package in R 3.5.1. Datasets were normalized to the baseline reading within a subject and day combination and a similar set of analyses to the VAS scores was conducted except with only subject specified as a random effect and no time effect included in the model. As per the analysis plan, results are expressed as changes from the baseline (Δ) to reduce possible variation resulting from the prolonged 16 h pre-treatment fast.

## 3. Results

### 3.1. Participants

Thirty participants completed all three treatments of the appetite trial. All participants were lean healthy males aged 24 ± 6 (18–40) years with a mean body mass index (BMI) of 23.1 ± 1.4 kg/m^2^ (20–24.9) (Table 2).

### 3.2. Composition of the Treatments

**Figure 3:**
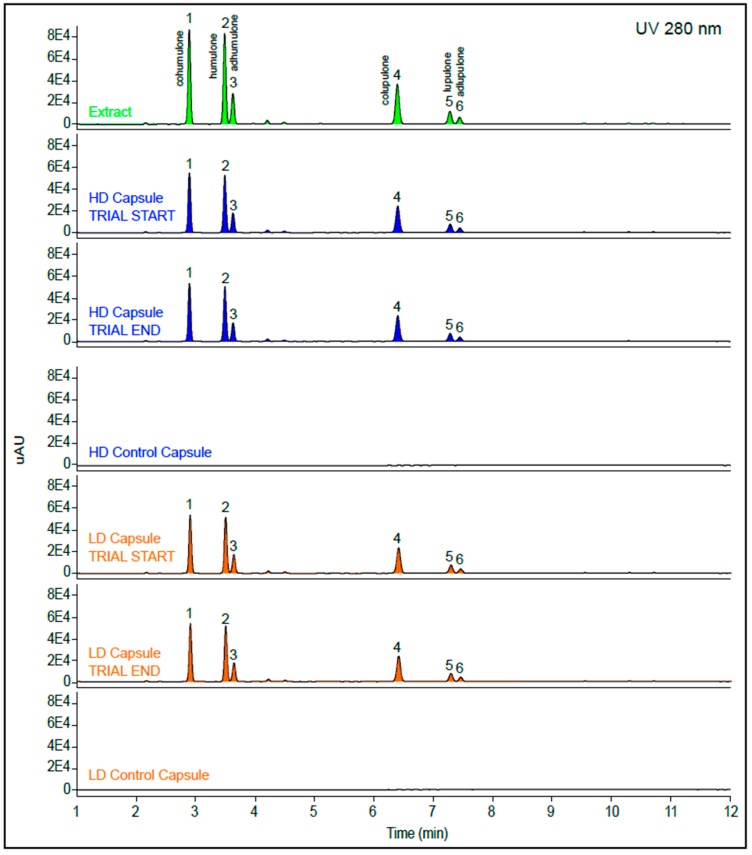
Liquid chromatography mass spectrometry (LCMS) chromatograms of the Amarasate^®^ hop extract, and high dose (HD), low dose (LD), and placebo capsules used in this study (1 mg/mL). Profiles of HD and LD capsules are shown at two time points, pre and post-trial. The UV response of the hop components for the capsules is 66.7% compared to the hop extract as expected, reflecting the 2:1 ratio of extract to canola oil used to prepare both HD and LD capsules. Trace peaks are 1. Cohumulone, 2. Humulone, 3. Adhumulone, 4. Colupulone, 5. Lupulone. 6. Adlupulone.

The capsules contained primarily bitter α- and β hops acids. The hops acid makeup was Cohumulone 21.08%, Humulone 22.25%, Adhumulone 8.15%, total α-acid 51.46%; Colupulone 19.71%, Lupulone 5.98%; Adlupulone 3.07%, and total β-acids 28.32%. No notable changes in chemical composition were observed over the course of the study (Figure 3), and further analysis of the HD capsules post-trial confirmed on-going chemical stability of the hop extract in canola oil 119 days post encapsulation (Supplementary Figure S1).

### 3.3. Appetite Ratings

**Figure 4:**
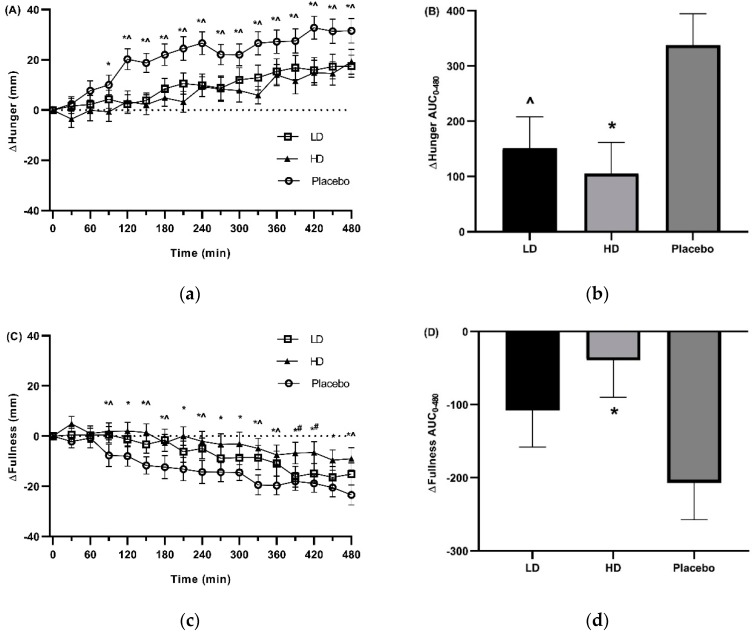
Visual analogue scale (VAS) results for hunger and fullness throughout the day in response to placebo, low dose (LD) and high dose (HD) Amarasate^®^ treatments. Changes (Δ) in the ratings of (**a**) hunger and (**c**) fullness from the 16 h (*t* = 0 min) to 24 h (*t* = 480 min) period of the 24 h fast. Histograms show AUCΔ0–480 min for Δ with respect to (**b**) hunger and (**d**) fullness from the 16 to 24 h period of the 24 h fast. Values are means ± standard error of the means (SEMs); *n* = 30. Treatment capsules were administered at *t* = 0 min and *t* = 240 min. Hunger and fullness exhibited a significant main effect for treatment *p* < 0.05 with evidence for a significant treatment x time interaction for hunger *p* < 0.05. Pairwise comparisons: HD vs. placebo, * *p* < 0.05, LD vs. placebo, ˄ *p* < 0.05, HD vs. LD, # *p* < 0.05.

**Figure 5:**
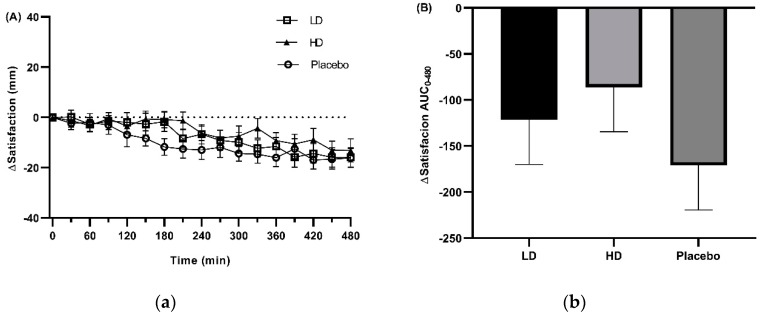

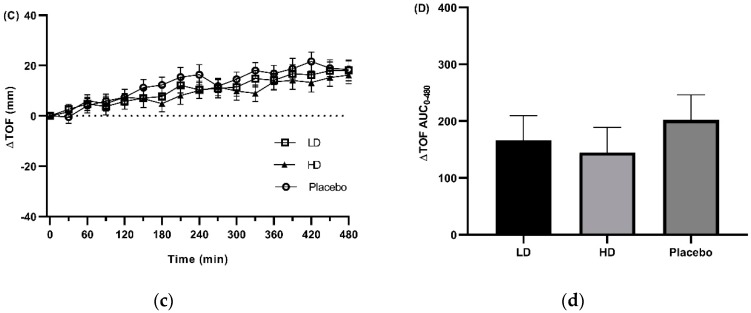
Visual analogue scale (VAS) results for satisfaction and thoughts of food (TOF) throughout the day in response to placebo, low dose (LD) and high dose (HD) Amarasate^®^ treatments. Changes (Δ) in the ratings of (**a**) satisfaction and (**c**) TOF from the 16 h (*t* = 0 min) to 24 h (*t* = 480 min) period of the 24 h fast. Histograms show AUCΔ0–480 min for Δ with respect to (**b**) satisfaction and (**d**) TOF from the 16 to 24 h period of the 24 h fast. Values are means ± standard error of the means (SEMs); *n* = 30. Treatment capsules were administered at *t* = 0 min and *t* = 240 min. Satisfaction and TOF did not so evidence for a treatment effect (*p* = 0.056, 0.211).

The mean changes (Δ) in VAS ratings for appetite related parameters of hunger, TOF, satisfaction, and fullness for the three groups are shown in Figure 4 and Figure 5. The placebo group recorded a 31.5 mm increase in VAS hunger ratings, a 23.4 mm decrease in VAS fullness ratings, 18.4 mm increase in VAS TOF ratings, and a decrease of 16.2 mm in VAS satisfaction ratings over the 16–24 h fasting period. These changes in these appetite measures are as expected and similar to previous examinations of fasting [34,49].

Mean changes (Δ) in VAS hunger ratings were lower compared with the placebo control for both the HD and LD treatment groups. Differences in excess of 10 mm were recorded for Δhunger for the HD treatment group relative to the placebo for all time points taken from *t* = 90 min onwards. This same magnitude difference was evident for all time points post *t* = 120 min for the LD treatment group. These changes in hunger were significant for main treatment effect when examining the entire 8 h treatment period (Figure 4a, *p* < 0.05). Pairwise comparisons confirmed reduced hunger for the HD treatment as compared to the placebo at time points *t* = 90, 120, 150, 180, 210, 240, 270, 300, 330, 360, 390, 420, 450, and 480 min, and for the LD treatment as compared to the placebo at time points t = 120, 150, 180, 210, 240, 270, 300, 330, 360, 390, 420, 450, and 480 min. At no time points were the HD and LD treatment groups significantly different from each other for Δhunger (Figure 4a). Figure 4b shows area under the curve (AUC) for Δhunger over the 480 min period between the start of monitored fasting (*t* = 0 min) and the final time point (t = 480 min) (ΔAUC_0–480 min_). A significant difference was observed between both the HD treatment and the LD treatment groups relative to the placebo control (Figure 4b, *p* < 0.05). No statistical differences were detected between the HD and LD treatment groups.

Overall there was evidence for a significant effect on fullness (Figure 4c, *p* < 0.05), which reflected VAS Δfullness increases of ≥ 10 mm relative to the placebo control for all time points from *t* = 120 to *t* = 480 min for the HD treatment and for only the *t* = 180 and *t* = 330 min time points for the LD treatment. Post hoc pairwise comparisons showed greater fullness at all time points from *t* = 90 min for the HD treatment, and time points *t* = 90, 150, 180, 240, 330, 360 and 480 for the LD treatment relative to the placebo control. Additionally, HD and LD group were significantly different at *t* = 390 and *t* = 420 min (Figure 4c). Figure 4d illustrates the AUC for Δfullness over the 480 min period between the start of monitored fasting (t = 0 min) and the final time point (*t* = 480 min) (ΔAUC_0–480 min_). A significant difference was observed between the HD treatment group relative to the placebo control (Figure 4d, *p* < 0.05), but not the LD treatment group relative to the control (*p* = 0.05). No statistically significant differences were detected between the HD and LD treatment groups.

Recorded VAS-rated changes in satisfaction and TOF (Figure 5) were similar but relatively reduced when compared with those of hunger and fullness. For VAS Δsatisfaction a 10 mm difference between the HD to placebo was observed at *t* = 180, 210 and 330 min. However, there was no evidence for a treatment effect (Figure 5a, *p* = 0.0556). VAS ΔTOF ratings did not differ by 10 mm at any time point between any of the treatment groups and there was no evidence for a treatment effect (Figure 5c, *p* = 0.2112). Histograms for Δsatisfaction and ΔTOF over the 480 min period between the start of monitored fasting (*t* = 0 min) and the final time point (*t* = 480 min) (ΔAUC_0–480 min_) are shown in 5b and 5d respectively (Figure 5b,d).

**Figure 6:**
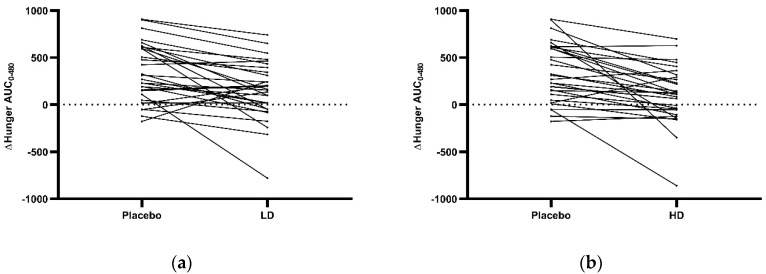
Individual area under the curve (AUC) results for Δhunger from the 16 h (*t* = 0 min) to 24 h (*t* = 480 min) period of the 24 h fast in response to placebo, low dose (LD) and high dose (HD) Amarasate^®^ treatments. Changes (Δ) in the AUC hunger for (**a**) placebo vs. LD and (**b**) placebo vs. HD.

Hunger responses for each participant were assessed by comparison of overall daily VAS ratings (ΔAUC hunger) during the LD and HD treatment days relative to the placebo treatment control day. Changes in individual measures for ΔAUC hunger over the 480 min period between the start of monitored fasting (*t* = 0 min) and the final time point (*t* = 480 min) (ΔAUC_0–480 min_) are shown in Figure 6a (placebo vs. LD) and Figure 6b (placebo vs. HD). Participants recorded similar responses to both treatments with 24 out of 30 showing decreased ΔAUC hunger for the LD and 25 for the HD relative to the placebo treatment control. Comparisons between the LD at HD treatment showed that 14 out of 30 had decreased ΔAUC hunger for the LD relative to the HD and 16 for the HD relative to the LD (data not shown). Post treatment nausea VAS scores did not exceed 10 mm in any of the treatment groups. Average water consumption was 1301.8 ± 133.0 mL, 1213.4 ± 172.5 mL, and 1234.9 ± 148.0 mL for the LD, HD, and placebo groups respectively, with no evidence of a significant difference between treatments. Liquid loose bowl movements were reported by three participants in the HD treatment group and by one participant in the LD treatment group and are an expected side effect from bitter compound regulation of colonic anion secretion [55].

## 4. Discussion

Traditional medicine records bitterness being used at times of food scarcity to reduce hunger and alleviate fast-related adverse effects [39,40], with recent research attributing this bioactivity to GI responses to bitterness [56]. Until now, bitter appetite suppressants have been examined as an adjunct to meal consumption, to either stimulate satiation by reducing energy intake at an ad libitum meal or to extend satiety from a fixed energy meal [57,58]. This current study highlights for the first time that prolonged anorexigenic effects of GI-targeted bitter compounds may be independent of food intake, and demonstrates the capacity for non-nutritive appetite suppressant co-therapy to enhance satiety during fasting. The observed daily changes in several appetite measures are highly relevant, occurring over a greater than 6 h timeframe and being in excess of the 10 mm considered to be biologically important [59]. Changes in hunger and fullness of this magnitude, and over this time period, may be reasonably expected to affect eating behavior [50], and hence may aid in fast compliance.

The results obtained in this current study are consistent with those observed by Deloose et al. 2017 when examining the effect of the potently bitter synthetic chemical denatonium benzoate on postprandial appetite measures. Deloose et al. [41] showed that GI delivery of denatonium resulted in a 15–20 mm reduction in VAS hunger ratings that persisted until their final time point 4 h post treatment. Here we report a similar magnitude change in hunger that persists for at least 4 h post treatment, although determining the exact duration of efficacy is difficult as a second treatment capsule was administered 4 h after the first. Combined, these results suggest that longer than 4 h of enhanced satiety may be possible from a single dose of a highly bitter gut-targeted appetite suppressant, and that even greater durations may be possible with repeat administration.

Appetite changes observed in the placebo group are generally in agreement with those of reported by Tinsley et al. 2018 [34] during their assessment through the same fasting period. As compared to Tinsley et al.’s results, the slightly decreased magnitude of changes in hunger and fullness reported here are likely because of the lower BMI range of participants in this current study (BMI 23.1 vs. 26.8 kg/m^2^). Although not assessed, the participants examined here would be expected to have low body fat, a characteristic that Tinsley et al. showed as a positive correlate for increased hunger during fasting. This current study also differed from Tinsley et al.’s research by prescribing a set fast commencement time, thus allowing for specific time of the day appetite assessments to be made. The reduction of lunchtime (12:00 h, *t* = 120 min) hunger observed here during Amarasate treatment has important implications for behavioral responses to fasting, with potential to improve both short term compliance and longer term adherence to the diet regime [60,61]. It is interesting that this 12:00 h time point exhibited the most pronounced change in daily hunger ratings, suggesting potential interactions between orexigenic meal time hunger signals and anorexigenic actions of the small intestinal targeted treatment [62,63]. Indeed, previous examinations of fasting show both spontaneous elevation of the orexigenic hormone ghrelin and increased VAS ratings for hunger immediately prior to 12:00 h [49,64], and that small intestinal delivery of bitter compounds stimulates GI peptide hormones that antagonize the pro-appetite actions of ghrelin [65]. Confirmation of the physiology underlying meal time appetite suppression and how this may affect fasting compliance warrant further investigation.

The mechanisms through which Amarasate affects hunger and fullness are most likely associated with anorexigenic gut peptides glucagon-like peptide-1 (GLP-1), cholecystokinin (CCK), and peptide tyrosine tyrosine (PYY). These peptide hormones are released during intestinal exposure to bitterness, and increase meal initiated satiation and enhance postprandial satiety [42,44]. It is therefore possible that these same peptide hormones may be responsible for the reduced hunger observed during this fasting trial. Indeed, the administration of GI peptides has been shown to reduce hunger during the fasted state [57,66,67]. Exogenous administration of GLP-1 induces a 25 mm reduction in hunger scores for fasted humans [68], infusion of physiological concentrations of PPY reduce composite appetite scores by a similar magnitude [69], and CCK injection shows potent suppression of acute hunger (> 50 mm) when given to fasted people [70]. These data suggest that the magnitude of hunger changes observed here could be explained by the actions of anorexigenic GI peptide secretion, and provides support for the conclusion that these hormones are likely mediators of the altered appetite ratings. However, as the relationship between anorexigenic gut peptides and appetite is complicated [71], we cannot preclude other mechanisms contributing to the observed effects, and it is possible that bitter compound regulation of intestinal motility and gastric accommodation may have contributed to the reported bioactivity [41,72].

Intermittent fasting is often associated with weight loss and hence is seen as an anti-obesity tool. However, fasting provides a series of health benefits that extend beyond reduction of body weight, including potential anti-aging effects and acute weight loss independent improvements in metabolic health [24,73]. It has been suggested that fasting related health benefits depend on metabolic switching that occurs 18–24 h into a water-only fast, highlighting that the fasting model tested may be appropriate to assess potential weight loss independent health benefits in this study’s population [74,75]. Given the positive result on appetite observed here, it may be prudent to assess metabolic switching as a surrogate marker for health during future fasting investigations. The role of IF for improved health rather than weight should not be overlooked, and may offer an avenue to affect meaningful health changes in a population that is typically not seen as requiring dietary intervention. Confirmation of the results observed here in overweight and obese individuals would be ideal, as this would allow the research to extend into the anti-obesity field. However, as fasting related hunger has been associated with body fat percentage [34], it may be advisable to examine shorter fasting periods when assessing appetite in this target population. Interestingly, cessation of prolonged fasting induces weight regain in overweight and obese individuals [76], further suggesting a potential role for anorexigenic agent co-therapy during intermittent fasting in this at-risk population.

The experimental design used in this study has several limitations that are worthy of discussion. Primarily, the decision to not strictly stipulate food consumption prior to and water consumption during the fasting period, and the differences in capsule weights that existed between the treatments.

The handling of water consumption and lead-in food intake during appetite trials is methodically problematic and no perfect solution exists. Indeed, the protocols on which this study’s design is based, Tinsley et al. 2018 [34] and Thivel et al. 2018 [49], take different approaches to both pre-fasting food prescription and allocation of water for consumption, while both examining appetite during a 24 h water-only fast. We decided to adopt Thivel et al.’s approach of asking participants to consume their usual amount of energy content prior to fasting and allowing water intake without specifying restriction. This determination was in part due to how closely Thivel et al.’s protocol reflected real-world fasting applications, and in part due to safety concerns stemming from possible treatment induced intestinal water loss and reduction of gastric capacity [55,72]. We acknowledge that this decision may have introduced additional variation to the appetite measures examined. The potential for different capsule weights to bias results was considered during the study design, and reflects a limitation imposed by the properties of the extract tested. It was decided that variation in capsule weight was an acceptable tradeoff for maintaining consistent formulation and ensuring stability of treatments [47,48]. As treatment capsules were taken directly from an opaque cup and treatments were administered one week apart, we believe it is unlikely that differences in capsule weight were apparent to the participants, however, we cannot preclude the possibility of induced bias. Particular strengths of this study include the fixed day of the week and time of day fasting protocol, the conformation of test extract stability pre and post study, and the selection of laboratory conditions that closely resemble real-world fasting applications.

In summary, we determined the efficacy of a bitter extract to regulate appetite during the 16–24 h period of a water-only fast and showed that the GI targeted delivery of a highly bitter hops flower extract can reduce hunger and increase fullness during the late stages of the fast. The results of this study indicate a potential role for anorexigenic agent co-therapy during fasting, and suggest the need for further studies examining the use of anorexigenic agents in different fasting methods and in overweight populations.

## Figures and Tables

**Table 1:** Composition of total daily treatments for the placebo control, the high dose (HD), and low dose (LD).

	Placebo	HD	LD
Amarasate^®^ Hops Extract (mg)	0	500	200
Canola Oil (µL)	250	250	100

**Table 2:** Characteristics of participants.

Characteristics of Participants	
Age, y (mean ± s.d, range, median (IQR))	24 ± 6, 18–40, 22 (8)
Ethnicity, Caucasian	19/30
Chinese	3/30
Persian	2/30
South American	2/30
New Zealand Maori	1/30
Sri Lankan	1/30
Filipino	1/30
Malaysian	1/30
Body weight, kg (mean ± s.d, median (IQR))	72 ± 7, 74 (8.5)
BMI, kg/m^2^ (mean ± s.d, median (IQR))	23.1 ± 1.4, 23.6 (2.2)

*n* = 30, s.d = standard deviation, BMI = Body Mass Index, IQR = Interquartile Range.

## Supplementary Materials

The following are available online at https://www.mdpi.com/2072-6643/11/11/2754/s1, Figure S1: Long term stability of HD capsule.
