# Supplementary material for: New Zealand Bitter Hops Extract Reduces Hunger During a 24 h Water Only Fast
Source: Nutrients. 2019 Nov 13;11(11):2754. doi: 10.3390/nu11112754 (PMC6893682; doi:10.3390/nu11112754)
Supplement: Supplementary file 1 [file nutrients-11-02754-s001.pdf]

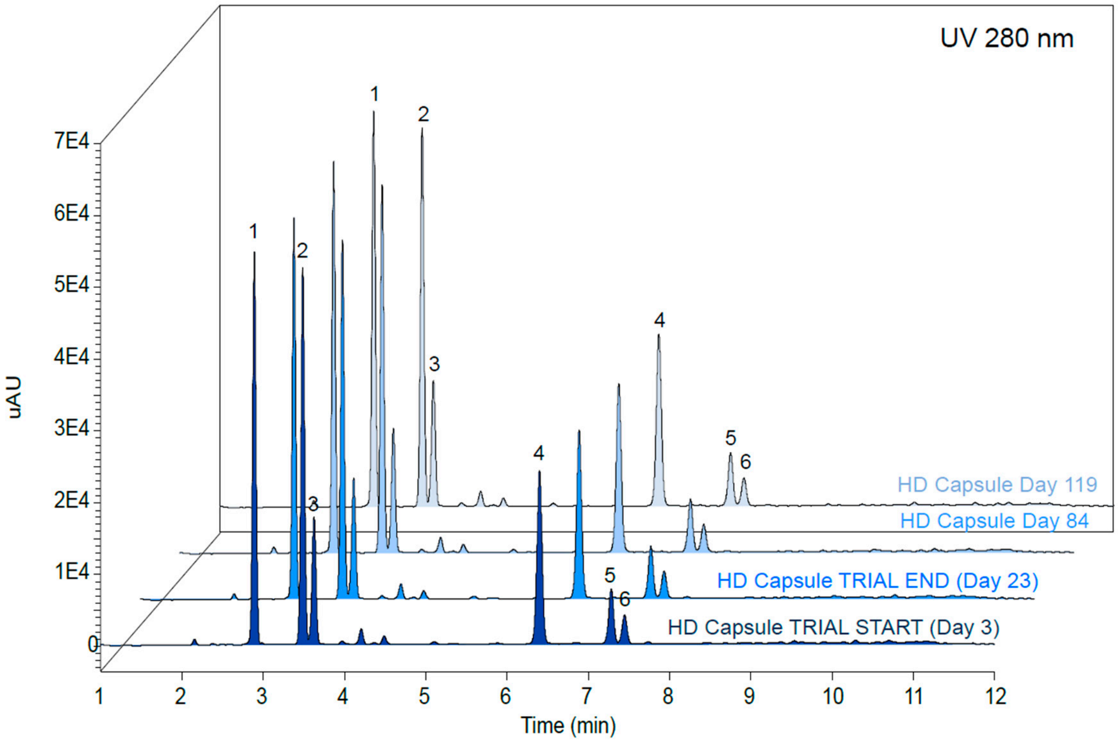

Supplementary Figure S1: Long term stability of high dose (HD) capsule. Liquid chromatography mass spectrometry (LCMS) chromatograms showing profiles of the HD Amarasate® capsules used in this study at four time points post encapsulation; pre-trial (three days post encapsulation), post-trial (23 days post encapsulation), 84 days post encapsulation, and 119 days post encapsulation
